# Human Guanylate Binding Proteins Potentiate the Anti-Chlamydia Effects of Interferon-γ

**DOI:** 10.1371/journal.pone.0006499

**Published:** 2009-08-04

**Authors:** Illya Tietzel, Christelle El-Haibi, Rey A. Carabeo

**Affiliations:** 1 Department of Natural Sciences, Southern University of New Orleans, New Orleans, Louisiana, United States of America; 2 Department of Microbiology and Immunology, University of Louisville Medical School, Louisville, Kentucky, United States of America; 3 Centre for Molecular Microbiology and Infection, Division of Cell and Molecular Biology, Imperial College London, London, United Kingdom; University of California Merced, United States of America

## Abstract

Chlamydiae are obligate intracellular pathogens that are sensitive to pro-inflammatory cytokine interferon-γ. IFN-γ-inducible murine p47 GTPases have been demonstrated to function in resistance to chlamydia infection *in vivo* and *in vitro*. Because the human genome does not encode IFN-γ-inducible homologues of these proteins, the significance of the p47 GTPase findings to chlamydia pathogenesis in humans is unclear. Here we report a pair of IFN-γ-inducible proteins, the human guanylate binding proteins (hGBPs) 1 and 2 that potentiate the anti-chlamydial properties of IFN-γ. hGBP1 and 2 localize to the inclusion membrane, and their anti-chlamydial functions required the GTPase domain. Alone, hGBP1 or 2 have mild, but statistically significant and reproducible negative effects on the growth of Chlamydia trachomatis, whilst having potent anti-chlamydial activity in conjunction with treatment with a sub-inhibitory concentration of IFN-γ. Thus, hGBPs appear to potentiate the anti-chlamydial effects of IFN-γ. Indeed, depletion of hGBP1 and 2 in cells treated with IFN-γ led to an increase in inclusion size, indicative of better growth. Interestingly, chlamydia species/strains harboring the full-length version of the putative cytotoxin gene, which has been suggested to confer resistance to IFN-γ was not affected by hGBP overexpression. These findings identify the guanylate binding proteins as potentiators of IFN-γ inhibition of *C. trachomatis* growth, and may be the targets of the chlamydial cytotoxin.

## Introduction

Infections of the obligate intracellular pathogens chlamydiae elicit an inflammatory Th1 response from the host resulting in their eventual clearance, with interferon-γ (IFN-γ) playing a prominent role in this process [1], [2], [3], [4]. Seminal studies from Byrne and colleagues have identified the IFN-γ-induced gene encoding for the catabolic enzyme indole 2,3-dideoxygenase (IDO) as the primary anti-chlamydial effector of IFN-γ [5]. IDO functions by cleaving the essential amino acid tryptophan, for which chlamydiae are auxotrophic, thus effectively starving the chlamydiae and inducing a growth arrest [6], [7], [8], [9], [10].

IFN-γ stimulation of cells *in vivo* and *in vitro* induces the transcription of numerous genes that cooperate to eliminate intracellular bacteria, and their roles in resolving bacterial infections are just beginning to be understood at the molecular level. There are other IFN-γ-induced genes that have not been characterized for bactericidal activity, and in addition to IDO may be required for the full manifestation of the anti-chlamydial function of this cytokine.

A prominent subset of these IFN-γ-induced genes includes a family of GTP-binding proteins, collectively termed large GTPases to which the 65 kD guanylate binding proteins (p65 GBPs) belong [11], [12], [13], [14], [15]. p65 GBPs represent well-conserved GTPases in vertebrates, and are highly induced by IFN-γ and less so by IFN-α/β [13]. There are five known GBP genes present in the human genome, and all are presumably induced by IFN-γ, owing to the presence of the interferon response element within their promoter sequences [13], [16]. hGBP1 and 2 have been demonstrated at the protein level to be induced by IFN-γ treatment. When maximally induced, the guanylate binding proteins can represent up to 20% of all the proteins induced by IFN-γ [15]. Its IFN-γ responsiveness and high levels of expression are highly suggestive of a role in effecting known IFN-γ-mediated downstream processes, such as immunity to viral pathogens or neovascularization during inflammation [17], [18], [19], [20], [21], [22]. In spite of its induction by IFN-γ, the role of hGBPs in the elimination of bacterial infections has not been thoroughly explored [23].

The mode of action of hGBPs is unknown, although it is well accepted that these proteins harbor a latent GTPase activity in its monomeric form that is markedly increased during oligomerization. Biochemical studies indicate that hGBP1 may hexamerize, with this form being the most potent in hydrolyzing GTP to GDP and GDP to GMP [15], [24], [25]. It has also been reported that stable expression of hGBP1 in HeLa cells resulted in the significant inhibition of vesicular stomatitis virus (VSV) and encephalomyocarditis virus (EMCV) replication, although its mode of action appears to be different for both viruses [19]. Inhibition of the VSV replication requires the N-terminal domain that contains a canonical GTPase domain, while the C-terminal domain, which has a high degree of helical structures and an isoprenylation (CAAX) domain, is necessary for limiting ECMV replication possibly through direct interaction with the ECMV nucleocapsid protein. Under IFN-γ treatment, hGBP1 was observed to localize to the Golgi apparatus [26]. However, this specific localization was not observed in ectopically expressed hGBP1, indicating an IFN-γ-responsive co-factor that mediates the association of hGBP1 with the Golgi apparatus. The role of hGBP's association with the Golgi apparatus in resistance to viral infections may not be important as ectopic expression of hGBP1 alone was sufficient in arresting VSV and ECMV replication.

In contrast to the limited knowledge of the antimicrobial effects of hGBPs, the importance of murine p47 GTPases in clearing chlamydia infection in mouse models has been reported and confirmed [27], [28], [29]. Several p47 GTPases have been shown to play a role in the restriction of *C. trachomatis* growth in murine cell lines, with the first evidence reported by Nelson *et al* describing the inhibitory effects on chlamydial growth by the p47 GTPase Iigp1 [29]. This study was followed by the identification of susceptibility loci in the mouse genome that harbored genes for Igtp and Irgb10, with the latter subsequently implicated directly on IFN-γ-mediated inhibition of chlamydial growth by associating with the inclusion membrane [30], [31]. In the same report, the murine homologue (Irga6) of Iigp1 was found to be dispensable in the resistance to chlamydia infection [28], a finding that is in conflict with that reported by Al-Zeer *et al* [32]. It is possible that a functional redundancy among these p47 GTPases exists and that the role of Iigp1 is masked *in vivo*. The p47 GTPase orthologues present in the human genome, IRGC and IRGM, are not inducible by IFN-γ [33], [34]. IRGC is also presumed to be a pseudogene. Interestingly, humans do not possess a p47 GTPase-based resistance. Thus, the importance of the findings in murine models of infection is unclear. The presence of hGBPs in the human genome, in conjunction with its lack of genes encoding p47 GTPases suggests that some function of the p47 GTPases in mice may be mediated by hGBPs in humans. To address this hypothesis, the relationship between hGBPs and chlamydial growth in cultured cells was characterized. We report that hGBPs link IFN-γ with its inhibition of chlamydial growth by acting as a potentiator, rather than an effector of the anti-chlamydial function of this cytokine.

## Materials and Methods

### Cell culture and reagents

HeLa 229 cells (CCL 1.2, ATCC) were grown as previously described [35]. Infections with *C. trachomatis* LGV443 (serotype L2), UW-3/CX (serotype D), and TW-5/OT (serotype B) (kindly provided by Dr. Ted Hackstadt, LICP, NIAID) at multiplicities of one were as previously described [35]. All experiments were performed at least three independent times.

### DNA constructs

TrueClones from OriGene encoding human GBP-1 (NM_002053) and GBP2 (NM_004120) were cloned into expression vectors with N-terminal c-Myc epitope tag (pCMV-Myc) or N-terminal hemagglutinin tag (pCMV-HA), kindly provided by Dr. Leigh Knodler (LICP, NIAID) for hGBP1 and hGBP2, respectively. Primers used were hGBP1 FWD EcoRI (5′ AAG AAT TCG GCA CGA GGG GAA CA 3′), hGBP1 REV Sal1 (5′AAG TCG ACT CTT TAG CTT ATG GTA CAT GCC 3′), hGBP2 FWD SalI (5′-AAG TCG ACC ATG GCT CCA GAG ATC AAC -3′), hGBP2 REV KpnI (5′-AAG GTA CCT TAG AGT ATG TTA CAT ATT GGC TCC AAT GA-3′), hGBP1 helical only FWD EcoR1 (5′-AAG AAT TCT CAA CGG GCC TCG TCT AGA-3′), hGBP1 helical only REV Sal1 (5′-AAG TCG ACT TAG CTT ATG GTA CAT GCC TTT CG-3′) were used to amplify hGBP1. PCR products were cloned into TOPO TA vectors (Invitrogen), and the insert removed by digestion with the appropriate restriction enzymes and ligated into either the pCMV-Myc or pCMV-HA vector. Integrity of the clones was verified by sequencing and protein expression. A dominant-negative mutant for GBP-1 (D184N) [36] was generated using the pCMV-Myc GBP-1 construct, the forward mutant primer 5′ GAC TTT GTG TGG ACA CTG AGA AAT TTC TCC CTG GAC 3′ and reverse primer 5′- TCT CAG TGT CCA CAC AAA GTC TGG GAA GAA GCT CAC -3′ and the GeneTailor Site-Directed Mutagenesis System (Invitrogen). The mutant clones were verified by DNA sequence analysis.

### Transfections and siRNA knockdown

Transfections were performed using the Fugene Transfection Reagent (Roche) per the manufacturer's suggestions, with slight modifications. SiRNA transfections were performed using the Ribojuice reagent, per the manufacturer's instructions. Optimized transfection conditions were as follows. Into 5×10^4^ HeLa cells plated on coverslips and grown in 0.2 ml Opti-MEM a 50-µl transfection mixture (30 nM siRNA consisting of three distinct siRNAs complexed, 2 µl Ribojuice, 48 µl Opti-MEM) was overlaid drop wise. siRNA used were from Ambion (human GBP-1 - #16708A ID 10835, #16708A ID 10930 and #16708A ID 11020; human GBP2 - Hs_GBP2_1 #S100425516, Hs_GBP2_2 #S100425523 and Hs_GBP2_4 #S100425537). To knockdown GBP-1 induced by human interferon-gamma (IFN-γ), cells were first incubated with siRNA transfection complexes. After 4 hours of incubation, the siRNA was replaced with cell culture medium containing 5 ng/ml IFN-γ. After 24 hours, cells were infected with *Chlamydia trachomatis* as indicated and the cytokine was kept in the medium during the whole cell culture period. One day later cells on the coverslip were processed for indirect immunofluorescence microscopy or immunoblots. Western blots for actin of whole cell lysates of equal cell numbers were used to monitor cytotoxicity of the siRNA treatment.

### Real time PCR

Expression of human Guanylate Binding Protein (GBP)-1, GBP2 and β-actin was assessed by isolating total RNA from 1×10^6^ HeLa cells using the TRIZOL reagent per the manufacturer's instructions, followed by reverse transcription ( 500 ng total RNA, Superscript III Reverse Transcriptase system (Invitrogen cat #18080-044), nonamer Random Primer 9 (New England Biolabs cat #S1254S)) and quantitative real-time polymerase chain reaction PCR. The QuantiTect primers for the gene GBP-1 (NM_002053; cat #QT01669885 or #QT00011641) with a predicted amplicon length of 96 bp and custom designed primers for GBP2 (FWD 5′-GAC CAA ATG TTC CAG AGG AAA TTA GGG GC-3′, REV 5′-AAT GTT CCC TGC TTG ACA TCT TCT TCT AAA GG-3′) were used. No cross amplification was observed between the primer pairs. For the gene ACTB (β-actin), cat #QT00095431 with a predicted amplicon length of 146 bp was used. The amplification of a specific product was independently verified further by analysis of the size of the end products of the PCR on a 2% ethidium bromide agarose gel and comparison with the corresponding predicted amplicon size. The quantitative analysis of expression levels of specific products was calculated and statistically evaluated using relative expression software tool from Pfaffl, MW *et al*. (Pfaffl MW, Horgan GW, Dempfle L. Relative expression software tool (REST) for group-wise comparison and statistical analysis of relative expression results in real-time PCR. Nucleic Acids Res. 2002 May 1;30(9):e36.). The expression of human β-actin was used as a reference gene to normalize the target genes of interest.

### Immunoblotting of whole cell lysates

Protein samples from whole cell lysates were analyzed by SDS-PAGE and Western blot, as described previously [35]. Primary antibodies used were anti-human GBP-1 (Oncogene, IM-1011), anti-actin (Sigma A3853), anti-β-tubulin (Sigma T4026), anti-Myc (Upstate clone 4A6) and anti-HA (Cell signaling clone 6E2). Secondary HRP-conjugated goat anti-mouse or anti-rabbit antibodies were from Pierce or Zymed.

### Indirect immunofluorescence microscopy

Preparation of samples for confocal imaging was performed as described previously [35]. Inclusions and transfected cells were visualized using an Olympus Fluoview confocal microscope (Olympus, Pittsburgh, PA) equipped with an argon and HeNe-G laser. Images were processed using Adobe Photoshop CS.

### Morphometric analysis and statistics

NIH Image J software (http://rsb.info.nih.gov/ij/) was used for the morphometric analysis of chlamydial inclusions. Differences in inclusion size under different growth conditions were analyzed by the Wilcoxon Rank Sum test (mcardle.oncology.wisc.edu/mstat). P-values of≤0.05 were considered statistically significant. For the statistical analysis of relative gene expression the Relative Expression Software Tool was used. The pairwise allocation function was employed with 2,000 iterations and p-values≤0.05 were considered statistically significant.

## Results

### hGBPs induction by IFN-γ correlated well with growth inhibition of chlamydia

**Figure 1:**
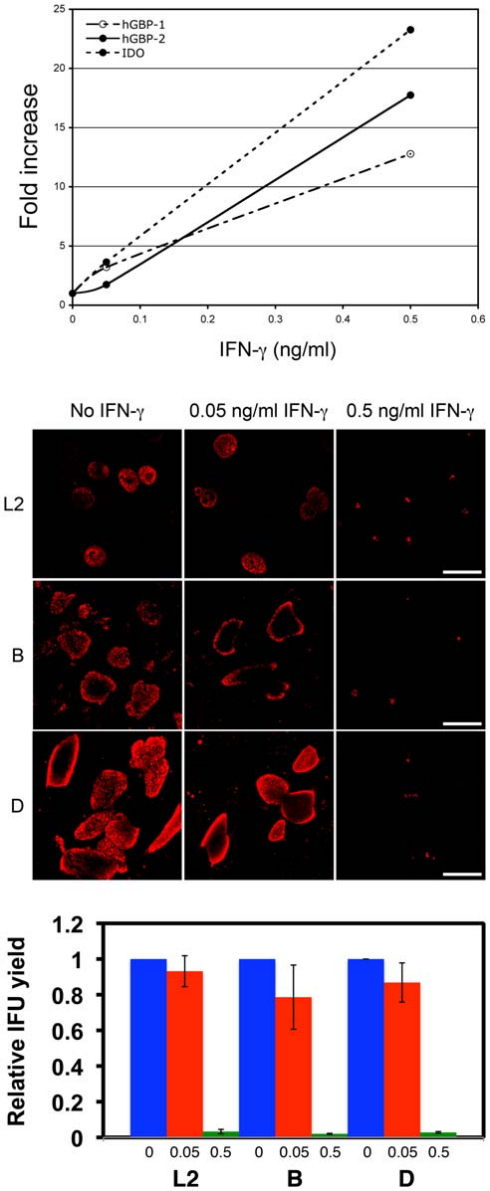
Induction of GBP under conditions that inhibit chlamydial growth. A) Transcriptional response of hGBP1 (open circle), hGBP2 (closed circle, solid line), and indole 2,3-dideoxygenase (closed circle, dotted line) to different doses of IFN-γ was monitored after 18 h treatment. Data are expressed as fold difference relative to mock-treated. B) Images of the inclusions of different *C. trachomatis* serovars from a parallel dose-response experiments are shown. Scale bars = 50 µm. C) IFU yields at 24 h p.i. (serovars L2, D) and 48 h p.i. (serovar B) for the different IFN-γ treatments.

hGBPs are induced by treatment with IFN-γ in other cell types, and a similar IFN-γ responsiveness was investigated in HeLa cells. Expression levels of hGBP1 and 2 after treatment with different doses of IFN-γ were examined by qRT-PCR at 24 h post-treatment. Figure 1 clearly demonstrated a mild, but noticeable induction of hGBP1 and 2 transcripts 24 h after treatment with 0.05 ng/ml dose of IFNγ, a dose that is inefficient in inhibiting the growth of various chlamydial strains and species as evaluated by microscopy and inclusion forming unit (IFU) assay. However, a robust increase was observed at a 10-fold higher dose (0.5 ng/ml). The induction of hGBP1, for which commercially available antibodies exist, could be readily observed *in situ* by immunofluorescence staining, as well as in Western blot (data not shown). At this IFN-γ concentration, which resulted in at least a 13-fold increase in transcript levels, clear inhibition of growth of *C. trachomatis* serovars L2, B, and D occurred in HeLa cells treated for 24 h prior to infection. At the identical IFN-γ concentration, IDO was induced by nearly 25-fold at the transcript level. Therefore, HeLa cells can induce hGBP1 and 2 in response to IFN-γ treatment, and increasing expression of hGBP1and 2, brought on by higher doses of IFN-γ correlated well with more efficient inhibition of growth of different *C. trachomatis* serovars.

### hGBP1 localizes to the chlamydial inclusion membrane

**Figure 2:**
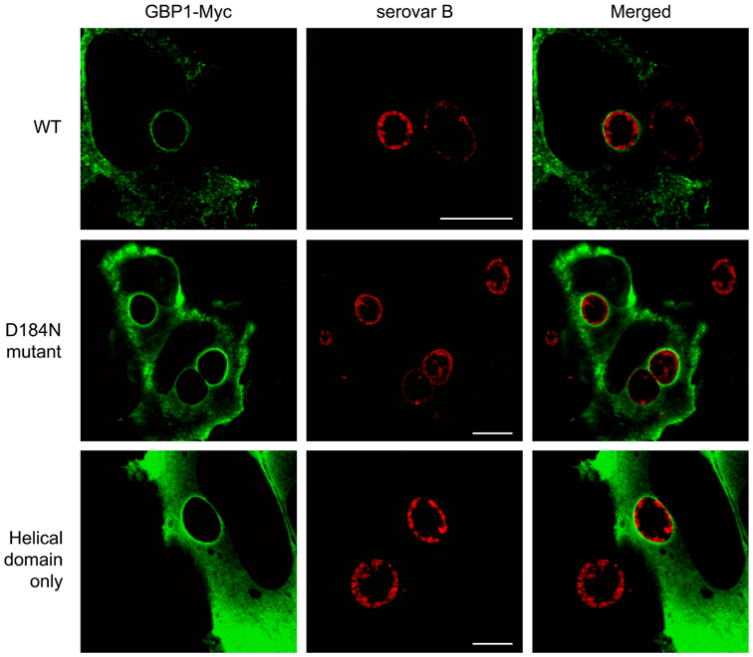
hGBP localizes to the inclusion membrane. HeLa cells were transfected with myc-GBP-1 expression construct (wt) and mutant derivatives, D184N dominant negative mutant, and helical domain only and infected with CtrB at 24 h post-transfection. Coverslips were processed 24 h post infection using for indirect immunofluorescence assays and visualized by laser confocal microscopy. Whenever possible, a neighboring untransfected cell harboring inclusions were shown.

The hGBPs are isoprenylated at their C-terminus via the CAAX domain, and the addition of this hydrophobic tag is thought to mediate association with membranous structures [21], [26], [36]. Therefore, localization of hGBP1 to the chlamydial inclusion was investigated by confocal microscopy. HeLa cells pre-transfected with the myc-tagged versions of the full-length or the C-terminal helical domain of hGBP1 were infected with *C. trachomatis* serovar B EBs. At 24 h p.i., the samples were processed for immunofluorescence microscopy. In Figure 2 chlamydia inclusion displayed rim-like staining patterns for both the full-length hGBP1 and the helical domain clones, indicating that the helical domain is sufficient to localize hGBP1 to the membrane. This is consistent with the CAAX isoprenylation motif localizing at the end of the helical domain. Chlamydia inclusions in untransfected cells on the same coverslip served as internal controls for the specificity of the antibody against the myc-tag. A similar rim-like staining pattern was observed for the HA-tagged full-length hGBP2 (data not shown).

### hGBP1 or 2 overexpression in HeLa cells delays *C. trachomatis* growth

**Figure 3:**
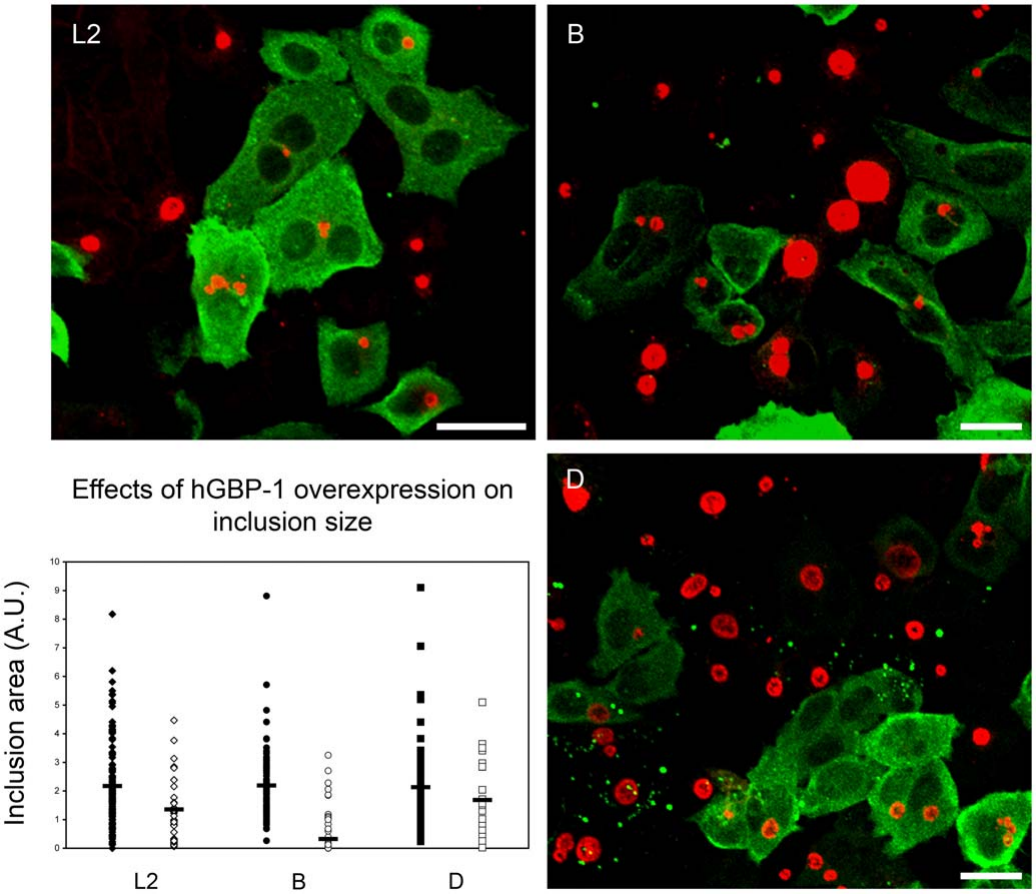
Overexpression of hGBP1 inhibits chlamydial growth. Cells overexpressing Myc-hGBP1 harbor smaller inclusions from Ctr serovars L2, B, and D. Samples were processed for immunostaining and confocal microscopy at 24 h post-infection. The size of the inclusion (red) in GBP-expressing cells (green) was measured using NIH ImageJ and compared to untransfected internal controls at 24 h p.i. Morphometric analysis showed a statistically significant difference (p<0.0001) in size of inclusions found in hGBP1-transfected (solid symbols) and untransfected cells (open symbols) for all serovars tested. Mean values are indicated by black horizontal bars. Data were analyzed using the non-parametric Wilcoxon test (two-sided).

The induction of hGBP expression by IFN-γ strongly implicates this GTPase family in antimicrobial defense. To study the potential anti-chlamydial activity of hGBP1 and 2 in the absence of any influence from other IFN-γ-induced genes, the genes encoding for hGBP1 and 2 were cloned under the control of the strong CMV promoter and downstream of either the Myc or HA tags. HeLa cells were transfected with myc-hGBP1 or HA-hGBP2, and at 24 h post-transfection, the cells were infected with *C. trachomatis* serovars L2, B, or D EBs at 1 MOI. At 24 h post-infection, the cells were stained for either the Myc or HA tag to identify cells expressing hGBP1 or hGBP2, respectively, along with an anti-L2 EB polyclonal antibody, which was sufficiently cross-reactive to recognize the other serovars used. Untransfected cells among the transfected ones were used as internal negative controls. Confocal images were obtained and each inclusion from either the transfected or untransfected cells was measured morphometrically using NIH ImageJ. Figure 3 shows a representative image of transfected and untransfected cells harboring developing inclusions of serovars L2, B, or D. Note the noticeably smaller size of inclusions found within cells overexpressing Myc-hGBP1. The differences in inclusion sizes were quantified by morphometric analysis and values expressed in arbitrary units (AU) are shown in Figure 3. Inclusions of all three *C. trachomatis* serovars growing in transfected cells were significantly (p<0.05, Wilcoxon Rank Sum Test) smaller (L2 – 1.461±0.89 AU, B – 0.365±0.29 AU, D – 1.725±1.63 AU) than inclusion in the untransfected cells (L2-2.177±1.26 AU, B – 2.254±1.56 AU. D – 2.182±1.47 AU). These data demonstrate that overexpression of hGBP1 is sufficient to produce a noticeable and statistically significant inhibition of chlamydial growth.

**Figure 4:**
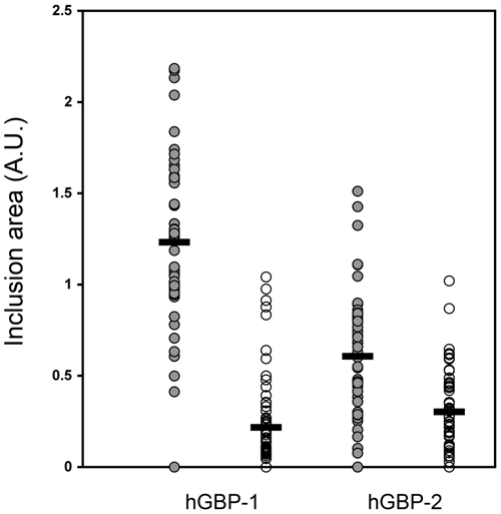
hGBP1 and hGBP2 have similar inhibitory effects on *C. trachomatis* serovar B. HeLa cells overexpressing hGBP1 or hGBP2 were infected with Ctr serovar B at 24 h post-transfection, and analyzed at 24 h post-infection. Mock-transfected cells from the same coverslips were used as internal negative controls. Mean values are indicated by black horizontal bars.

Because of the pronounced sensitivity of serovar B to hGBP1 overexpression, this serovar was used for subsequent studies. The effects of overexpression of hGBP2 on the growth of Ctr serovar B inclusions were evaluated. Indeed, a noticeable decrease in mean size was consistently observed for serovar B inclusion found in cells overexpressing hGBP2, indicating that like hGBP1, hGBP2 may have anti-chlamydial function (Figure 4).

Inhibition of *C. trachomatis* growth by hGBP1 requires the GTPase activity.

**Figure 5:**
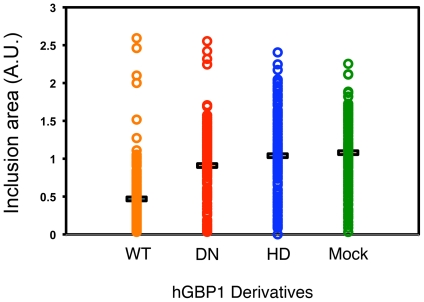
Anti-chlamydial activities of hGBP1 require the GTPase region. HeLa cells were transfected with constructs encoding either wild type GBP-1 (wt), a dominant negative mutant (D148N). The cells were infected with Ctr serovar B and processed for confocal microscopy at 24 h p.i. Inclusions from the cells expressing the wild-type form of hGBP1 were measured and statistically compared with the cells expressing the D148N mutant (DN), the helical domain only (HD), and mock-transfected using Mann-Whitney test. Mean inclusion sizes are indicated by horizontal bars. P-value<0.005.

Because of the similar effects of hGBP1 and hGBP2 on Ctr serovar B, functional analyses were focused on hGBP1. A structure/function study was performed to determine the requirement for the GTPase domain. Mutant derivatives of hGBP included the deletion of the entire GTPase domain, and a D184N point mutant that has been reported to lack the GTPase activity [36]. At 24 h post-transfection, the cells were infected with serovar B EBs, and the chlamydia was allowed to grow for 24 h. Ctr serovar B was chosen for its sensitivity to hGBP1 overexpression. The samples were processed for immunofluorescence imaging, and the area of each inclusion area was morphometrically measured. The deletion mutant, and the subtler D184N point mutant lost the ability to inhibit chlamydia growth (Figure 5).

### hGBP1 and 2 knockdown partially relieved the chlamydia growth inhibition by IFN-γ

**Figure 6:**
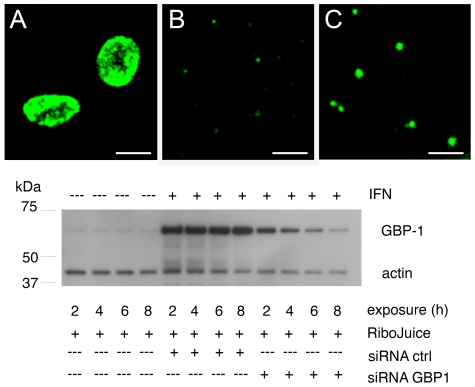
Knock-down of hGBP1 partially restores growth of chlamydia inclusions. Untreated (A) or IFN-γ treated HeLa cells were exposed to scrambled (B) or anti-hGBP1 (C) siRNA. At 24 h post-transfection, the cells were infected with Chlamydia trachomatis serovar B, and incubated for a further 24 h prior to processing of samples for confocal microscopy. The total transfection and treatment times were 48 h. Scale bar = 20 µm. Inclusion areas of mock and hGBP1-depleted samples were measured. Statistical significance (p<0.002) was determined using the Wilcoxon Rank Sum test (two-sided). The bottom panel demonstrates the optimization of the 48-h transfection and co-treatment protocol to achieve the maximum reduction in hGBP1 while minimizing cytotoxicity.

To further confirm the potentiating properties of overexpressed hGBP1 in the context of IFN-γ function, HeLa cells were transfected with siRNAs for hGBP1 to reduce the level of the protein in IFN-γ-treated cells. At 48 h post-transfection, when the level of hGBP1 protein was at its lowest relative to untransfected cells during a 24-h treatment with IFN-γ (5 ng/ml), the cells were infected with *C. trachomatis* serovar B EBs. Ctr serovar B was chosen for its relatively higher level of susceptibility to IFN-γ treatment. It should be noted that IFN-γ was present 24 h prior to infection and for an additional 24 h during infection. The cells were processed for immunofluorescence confocal microscopy and morphometric measurement of inclusions. Figure 6 shows representative confocal images of inclusions in mock-transfected and siRNA-transfected cells under identical magnification in the absence (A) or presence (B and C) of IFN-γ. In addition, the level of knockdown of hGBP1 at the experimental conditions used (lane 11 of 12) is shown by the accompanying Western blot. The size of the inclusions were measured and statistically analyzed for any differences that may have resulted from siRNA depletion of hGBP1. Measurement of at least 50 inclusions from each group yielded a statistically significant difference (p<0.0003, Wilcoxon Rank Sum test) in inclusions sizes among the mock-transfected (0.255±0.106 AU) and hGBP1 siRNA-transfected (0.742±0.233 AU) HeLa cells. Untreated cells served as a control for the potency of the stock of IFN-γ used. A similar result was obtained for Ctr serovar L2 albeit with a more moderate difference between mock and hGBP1-depleted groups (data not shown). Better growth was obtained when the maximal induction by IFN-γ of hGBP1 was prevented by siRNA knockdown.

### hGBP1 potentiated the anti-chlamydial activity of IFN-γ

**Figure 7:**
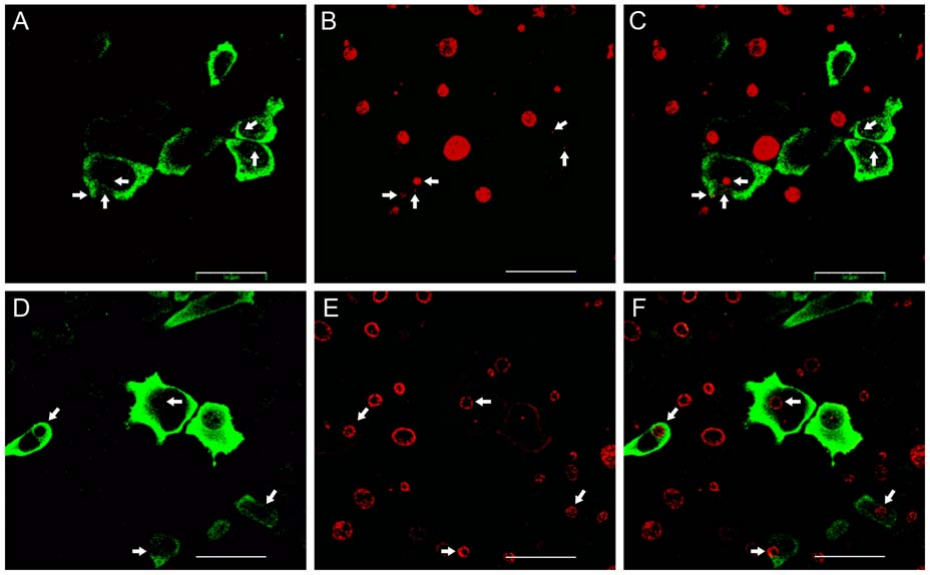
hGBP1 potentiates the anti-chlamydial effects of IFN-γ. Cell overexpressing myc-hGBP1 (A-C) or myc-hGBP D148N (D-F) were treated with a sub-inhibitory concentration (0.05 ng/ml) of IFN-γ at the time of infection with Ctr serovar L2 at MOI = 1 and processed for microscopy at 24 h p.i. White arrows indicate inclusions within transfected cells. Scale bar = 50 µm.

The anti-chlamydial activity associated with hGBP1 and 2 overexpression was relatively modest when compared to that of IFN-γ treatment. However, their robust induction by IFN-γ suggests a potential role in mediating some function of this cytokine. Rather than being a direct effector of IFN-γ with regards to chlamydia growth inhibition, we hypothesized that hGBPs may potentiate the effects of IFN-γ. To test this hypothesis, HeLa cells overexpressing myc-hGBP1 were treated with a sub-inhibitory dose of IFN-γ (0.05 ng/ml) at the time of infection (at 18 h post-transfection) with Ctr serovar L2. Thus, cells were not pre-treated with IFN-γ. This strain was chosen for its relative resistance to hGBP1 overexpression (Figure 3). Thus, any inhibition of Ctr L2 growth due to the concerted action of hGBP1 overexpression and the sub-inhibitory concentration of IFN-γ would be considered significant. In untransfected cells, morphologically mature inclusions could be seen in the presence of IFN-γ at a dose (0.05 ng/ml) that has been shown to be insufficient to negatively affect chlamydial growth (Figure 1). Interestingly, noticeably smaller inclusions could be observed in cells overexpressing hGBP1 (Figure 7 A-C), while no noticeable change in sizes of those in cell expressing the D148N mutant were seen. The level of responsiveness of the cells was found to be unaltered by exposure to the transfection reagent (Supplemental Figure S1). Furthermore, the percentage of cells infected with chlamydia and transfected with hGBP1-myc in IFN-γ treated (10%) and mock-treated (42%) populations were significantly different, suggesting that the combination of hGBP1 overexpression and the sub-inhibitory concentration of IFN-γ cooperated to kill and degrade internalized EBs, rendering them undetectable by immunofluorescence. Taken together, the data indicate that the sub-inhibitory concentration of IFN-γ could be sufficient in inhibiting chlamydial growth when accompanied by hGBP1 overexpression.

A correlation between sensitivity to guanylate binding protein overexpression and presence of the full-length cytotoxin gene.

**Figure 8:**
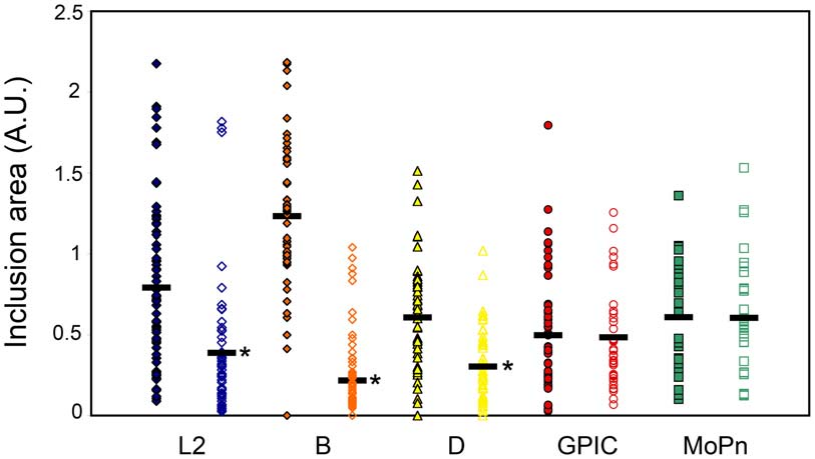
Correlation between sensitivity to hGBP1 overexpression and the presence of full-length cytotoxin. HeLa cells transfected with hGBP1 were infected at 24 h post-transfection with *C. trachomatis* L2, B, or D strain, *C. caviae* serovar GPIC, and *C. muridarum* serovar MoPn. The infection was terminated at 48 h post transfection and areas of inclusion in transfected (closed) and untransfected (open) cells were measured as above. hGBP expression yielded statistically significant differences in inclusion size, except for hGBP1 expression in GPIC or MoPn-infected cells. * indicate p<1×10^−5^ in the two-sided Wilcoxon test.

A report by Nelson *et al* demonstrated that gamma-irradiated *C. muridarum* EBs conferred IFN-γ resistance to *C. trachomatis* serovar D during growth in murine cells [29]. This apparent “acquired” resistance was attributed to the cytotoxin in *C. muridarum* EBs. With hGBPs possessing anti-chlamydia activities, a survey of different strains and species of chlamydia was conducted. Strains and species chosen were *C. trachomatis* serovars L2, B, D, *C. caviae* GPIC strain, and *C. muridarum*. Ctr L2 lacks the cytotoxin gene; Ctr B and D encode a partial cytotoxin; *C. caviae* and *C. muridarum* possess a full-length cytotoxin gene. Using a similar morphometric analysis described above, an interesting correlation was observed with regards to the presence of the cytotoxin (Figure 8). The species with full-length cytotoxin genes were relatively resistant to the hGBP overexpression. In contrast, the partial cytotoxin gene was not sufficient to confer the same level of resistance, as shown by retained sensitivity of Ctr B and D. Expectedly, the Ctr L2 serovar remained susceptible to hGBP overexpression. Also, rim-like staining by hGBP1 was inconsistently observed for *C. muridarum* inclusions (data not shown). Because of the irregular morphology of the *C. caviae* inclusions, it was difficult to make any conclusion as to the inclusion membrane localization of hGBP1. Based on the results obtained for *C. muridarum*, the intact cytotoxin may counteract the anti-chlamydial effects of hGBP overexpression by displacing them from the inclusion membrane. However, the influence of other polymorphisms cannot be discounted at this time.

## Discussion

Interferon-γ is required for the timely clearance of *C. trachomatis* infection [37], [38]. Its anti-chlamydial mechanism has been attributed to the tryptophan catabolizing enzyme indole 2,3 dideoxygenase (IDO) [5], [7], [39]. In culture, the addition of excess tryptophan in the growth media that was supplemented with IFN-γ at inhibitory concentrations resulted in the complete rescue of chlamydial growth [5], [7], [39]. While IDO certainly is essential in clearing chlamydia infection, it is likely that other anti-chlamydial effectors of IFN-γ exist to cooperatively eliminate chlamydia infections *in vivo*.

We have identified a family of proteins that appears to potentiate, rather than directly mediate the anti-chlamydial function of IFN-γ. They are the p65 guanylate binding proteins (GBP), of which five isoforms have been identified in humans [34]. hGBPs constitute approximately 20% of the IFN-γ induced proteins [15], [25], suggestive of its function in IFN-γ-mediated elimination of microbial/viral infections. hGBP1 has been implicated in the resistance to VSV and ECMV infection, with the anti-viral mechanism of GBP-1 requiring the GTPase function [19], [22]. Thus, one possible mechanism is the efficient hydrolysis of GTP to GMP, via a GDP intermediate [24], and this GTPase activity is greatly enhanced *in vitro* when GBPs hexamerize [40]. In this report, we demonstrate the loss of anti-chlamydial activity of hGBP1 when a mutation (D148N) that abrogated the GTPase activity was introduced. Thus, growth inhibition of chlamydia by hGBP1 and 2 could be due to their functions in signalling and/or guanine nucleotide depletion.

An unexpected finding was the ability of hGBP1 to potentiate the anti-chlamydial effects of IFN-γ even at sub-inhibitory concentration of the cytokine. It should be noted that while the dose of IFN-γ used was insufficient to arrest chlamydial growth, a transcriptional response to IFN-γ treatment were still induced, as demonstrated by the 4-fold increase in IDO induction in the presence of IFN-γ (0.05 ng/ml). At this dose, hGBP1 and 2 were also induced by 2 to 3 fold. Assuming cooperation between IDO, hGBP1 and 2, it would appear that the respective levels of induction with 0.05 ng/ml IFN-γ were not sufficient to inhibit chlamydial growth. However, by artificially increasing hGBP1 levels via ectopic overexpression, the addition of the sub-inhibitory concentration of IFN-γ became sufficiently potent in negatively affecting the growth of Ctr serovar B. This mode of action is reminiscent of a potentiator, rather than an effector. Also, the relatively early induction of hGBPs in cultured cells after exposure to IFN-γ is consistent with this potentiating function by making chlamydia more susceptible to the subsequent assault of the many anti-bacterial effectors of IFN-γ expressed.

The mode of action of hGBP1 and 2 are not known. However, we did observe the localization of hGBP to the inclusion, and we speculate that this localization mark the inclusions for interaction with degradative compartments of the host cell. Interestingly, hGBP1 was found to localize, albeit inconsistently to LC3-positive autophagosomes of uninfected cells (Tietzel and Carabeo, unpublished observation). The localization of hGBP1 to the chlamydial inclusion during conditions of overexpression may render this parasitophorous vacuole recognizable to the autophagy machinery, as has been shown by Al-Zeer *et al* with the murine Irga6 [32]. Indeed, related murine p47 GTPases, which are not found in the human genome, are known to be involved in autophagy and other mechanisms that promote the interaction of the phagosome with degradative compartments within the cell [41], [42]. Irga6-mediated alteration of the chlamydial inclusion may result in the non-fusogenicity of this pathogen-defined organelle with sphingomyelin-containing vesicles originating from the trans-Golgi. Indeed, Nelson *et al* observed the inhibition of SM traffic to the inclusion [29].

In addition, localization of a subset of murine p47 GTPases (Irgb10 and Irga6) to the inclusion correlated with susceptibility to IFN-γ with *C. trachomatis* inclusions, but not of the more resistant *C. muridarum* positive for the GTPase proteins [32]. This observation led to the speculation that localization of the p47 GTPases could be determined by the cytotoxin content of the chlamydia.

While humans lack the homologous genes, the p65 GTPases to which hGBPs belong may possess functions similar to the murine p47 GTPases. If hGBPs are indeed functioning within the context of the anti-chlamydial function of IFN-γ, then their depletion would be expected to have noticeable effects on the efficiency of chlamydial inhibition by this cytokine. Indeed, knockdown of hGBP1 and hGBP2 levels by siRNA in the presence of IFN-γ visibly attenuated the anti-chlamydial effects of this cytokine, further implicating an anti-chlamydial role for these GTPases. The depletion experiments did not lead to the complete restoration of inclusions to normal size, but this result is not surprising given the number of potential anti-chlamydial effectors induced by IFN-γ that still remained. However, the visible attenuation of growth inhibition upon their depletion indicates a prominent role for these GTPases in resolving chlamydia infection – an observation consistent with a potentiating mode of function for hGBPs. Further supporting the potentiator model is the demonstration of growth inhibition of inclusions growing in cells overexpressing hGBP1 and concomitantly treated with sub-inhibitory concentration (0.05 ng/ml) of IFN-γ.

It should be noted that the proposed potentiator function of hGBPs in IFN-γ-mediated inhibition of chlamydial growth does not necessarily contradict the previous findings regarding indole 2,3 dideoxygenase (IDO) as the major effector, as hGBPs may sensitize chlamydia to tryptophan starvation. Possible mechanisms may include prevention of nutrient acquisition through alterations to inclusion properties. GTP depletion may also cooperate with tryptophan depletion as guanosine synthesis is linked to tryptophan synthesis via the phosphoenolpyruvate carboxylase (PEC) enzyme, which determines the levels of metabolic intermediates within the tricarboxylic acid cycle [43], [44]. Modulation of the levels of these intermediates has consequences on the biosynthesis of downstream metabolites, such as guanosine and guanine nucleotides, which are derived from α-ketoglutarate [45]. Both scenarios could be alleviated by the addition of excess tryptophan in the growth medium to bypass the potentiating effects of hGBP.

One interesting observation that was made was the relative resistance to hGBP overexpression of chlamydia species that encode the full-length cytotoxin. This piece of data implies that the cytotoxin, which is homologous to clostridial toxins that target host GTP-binding proteins, may neutralize hGBPs. Because the cellular target for the toxin, or indeed its function have yet to be identified, this scenario becomes an intriguing possibility. Nelson *et al* have already implied a functional relationship between the cytotoxin and a member of the murine p47 GTPases, although other polymorphisms within the genome could account for the difference in susceptibility to IFN-g [29]. If indeed true, our data would lend further support to the GTPase-inactivating function of the putative chlamydia toxin.

This report as a whole represents the first characterization of hGBPs in the context of chlamydia infection. By identifying hGBPs as potentiators of IFN-γ function, we introduce a new perspective to IFN-γ-mediated suppression of chlamydial growth – that the collection of IFN-γ-induced genes with anti-chlamydial functions could be classified as potentiators and effectors, with hGBPs belonging to the former class, and IDO to the latter. Our ongoing studies on the functions of hGBPs in chlamydia pathogenesis should provide some insight into the mechanisms of IFN-γ-mediated clearance of bacterial infection in humans.

## Supporting Information

Figure S1 Transfection reagents do not dampen the anti-chlamydial function of IFN-γ HeLa cells were exposed to mock, Fugene, or Ribojuice transfection cocktail for 24 h. After removal of the transfection reagents, the cells were infected with C. trachomatis serovar L2 and immediately followed by treatment with IFN-γ (Mock - blue; 0.05 ng/ml - red; 0.5 ng/ml - green). At 24 h p.i., IFU yield was determined for each group and expressed as values relative to the mock-treated group.
(1.22 MB TIF)
